# Self-reported acute pesticide intoxications in Ethiopia

**DOI:** 10.1186/s12889-016-3196-5

**Published:** 2016-07-15

**Authors:** Amare W. Nigatu, Magne Bråtveit, Bente E. Moen

**Affiliations:** Department of Global Public Health and Primary Care, University of Bergen, Bergen, Norway; Centre for International Health, University of Bergen, Bergen, Norway

**Keywords:** Acute pesticide intoxications, Self-reported symptoms, Flower farm workers

## Abstract

**Background:**

Pesticide exposure is an important public health concern in Ethiopia, but there is limited information on pesticide intoxications. Residents may have an increased risk of pesticide exposure through proximity of their homes to farms using pesticides. Also the pesticide exposure might be related to employment at these farms. This study investigated the prevalence of acute pesticide intoxications (API) by residence proximity to a nearby flower farm and assessed if intoxications were related to working in these farms or not.

**Methods:**

A cross-sectional survey involving 516 persons was conducted. Participants were grouped according to their residence proximity from a large flower farm; living within 5 kilometers and 5–12 kilometers away, respectively. In a structured interview, participants were asked if they had health symptoms within 48 h of pesticide exposure in the past year. Those who had experienced this, and reported two or more typical pesticide intoxication symptoms, were considered as having had API. Chi-square and independent t-tests were used to compare categorical and continuous variables, respectively. Confounding variables were adjusted by using binomial regression model.

**Results:**

The prevalence of API in the past year among the residents in the study area was 26 %, and it was higher in the population living close to the flower farm (42 %) compared to those living far away (11 %), prevalence ratio (PR) = 3.2, 95 % CI: 2.2-4.8, adjusted for age, gender & education. A subgroup living close to the farm & working there had significantly more API (56 %) than those living close & didn’t work there (16 %), adjusted PR = 3.0, 95 % CI: 1.8-4.9. Flower farm workers reported more API (56 %) than those not working in the flower farm (13 %,), adjusted PR = 4.0, 95 % CI: 2.9-5.6.

**Conclusion:**

Our study indicates a 26 % prevalence of self-reported symptoms attributable to API. The residents living closer than 5 kilometers to the flower farm reported significantly higher prevalence of self-reported API than those living 5–12 kilometers away. This increased risk of API was associated with work at the flower farm.

## Background

Pesticides are widely used in the agriculture sector globally to control pests, and in less developed countries the use of pesticides is increasing [1]. Spraying of pesticides to protect crops may cause human exposure during spraying, followed by adverse health effects [2–4]. This is an important public health concern in developing countries [3]. Several studies have shown that occupational exposure to pesticides is common among farm workers, resulting in high prevalence of acute pesticide intoxication (API) [4–7]. According to a survey of self-reported minor poisoning from pesticides, there could be as many as 25 million agricultural workers in the developing world suffering an episode of pesticide poisoning each year [8]. In Ethiopia over 85 % of the population depend on agriculture, and the activity is mainly characterized by small-scale farming. The use of pesticides in agriculture has increased dramatically in Ethiopia the last decades. The increased demands for productivity and the expansion of commercial farms, especially floriculture, are the prime factors. Floriculture is growing at a very fast rate involving tens of thousands of workers [9]. The cultivation of roses in these farms is highly dependent on extensive use of pesticides. The most common pesticides used in Ethiopia include organophosphates, carbamates and to some extent organo-chlorines [10]. The flower farms are usually located in close proximity to the houses of the rural community. Pesticides sprayed in the flower farms as well as on crops at small farms may increase pesticide exposure of the population. A study by Ward, et al. in USA, suggested that rural residents could be exposed to agricultural pesticides through proximity of their homes to crop fields. The study indicated that six herbicides, used almost exclusively in agriculture, were detected in 28 % of the homes [11]. In Ethiopia, most workers in the flower farm reside in nearby villages, and they might be exposed to pesticides at their workplace.

To our knowledge, there is limited information about the magnitude of API in Ethiopia. Those who use pesticides, i.e., in households, smallholder private farmers as well as flower farms workers, could all experience API. We speculated that there could be differences in API experience and related adverse health effects between different population groups according to proximity to flower farms (living close vs. far away) as well as being employed in the flower farm or not. Persons living close to the flower farms or working at these farms might have greater access to the pesticides and be more exposed to these substances. The objectives of the present study were to: i) determine the prevalence of API among residents in an area where a flower farm is located; ii) study the relationship between API and residential proximity to the flower farm and; iii) assess if the prevalence of API is related to the work in the flower farm.

## Methods

### Study design and study setting

A cross-sectional study involving 516 households was conducted from August to September 2014, in Ethiopia. One person from each of the selected households, usually the head of the household, was interviewed using a structured interview guide. When the household head was unavailable, the interviewer asked for consent to interview the first adult over 18 years met in the household.

### Study area and study population

The study area comprised a total of 1025 villages in a district where one of the largest flower farms in Oromia region of Ethiopia, involving over ten thousand workers, is located. Lists of villages and households in this area were obtained from the local authorities as well as from a research project run by researchers from Bergen and Addis Ababa Universities [12]. For the purpose of this study, we defined two observational groups; residents living close vs. far away; /<5 & 5-12/ kilometers from the flower farm, respectively. We decided to have 5 kilometer cut-off point based on our observations of the study area. Most of the working population in the flower farm lives within a distance of 0–5 kilometer from the flower farm while those living within 5–12 kilometer are mainly involved in small-scale farming. The12 kilometer cut-off point was chosen to exclude the residents living in the villages right after the 12 kilometer mark who mostly work at a pesticide factory.

The flower farm grows roses inside plastic greenhouses. The small private farms in the area mostly grow cereal crops such as maize, wheat, and sorghum. Pesticides are widely used both for rose cultivation and for growing cereal crops, though there are differences in the types and intensity of pesticides used [10].

### Sampling technique and sample size

Cluster sampling technique was used, where a village, which is the smallest administrative unit, was considered as a cluster. The number of households in a village varied considerably, ranging from 40 to 100 households (200 to 500 inhabitants) per village; and the villages closer to the flower farm were much larger than those located far away. All the villages, which were located within 0–12 kilometer from the flower farm, formed the sampling frame; a total number of 68 villages, i.e., 23 & 45 villages located < 5 & 5–12 kilometer away, respectively. Based on the prevalence of excessive sweating (25 %), a typical symptom of organophosphate intoxication reported in a previous study [13], we calculated a sample size of 520 households needed to achieve a statistical power of 80 %, at a significance level of *p* < 0.05. According to this calculation a total of 11 (4 close & 7 far) out of 68 villages were randomly selected to get the required number of households. All the households in these selected villages were invited to participate in the study.

### Interview

Interviews were performed from 9:00–17:00 using a structured interview guide developed from similar previous studies done elsewhere in English language [3, 14–16]. The interview guide was translated from English to the local language (Afan Oromo) and vice-versa. A pretest was conducted among ten households in the area, which were excluded from the final analysis. Some minor changes, such as redefining the job categories to accommodate all job types, were made before the interview guide was finalized.

Exposure to pesticides may occur among agricultural workers in open fields and in greenhouses through occupational exposure, and among persons using pesticides to control house pests. Moreover, although a particular occupation does not actually use pesticides themselves, the presence of pesticides in the working environment constitutes potential occupational exposure for them [17].

As shown in Table 1, the questions were on socio-demographic information, current job, work experience, pesticide use, experienced health problems within 48 h of exposure to pesticides in the last year, and whether the exposure to pesticides occurred through occupational exposure in the case of flower farm workers and small-scale farmers or pesticide application for household pest control. The respondents, who explained a plausible description of exposure to pesticides; and reported to have experienced health problems within 48 h of the exposure once or several times the past year, were asked to state the health symptoms they had. The interviewers then ticked off the symptoms they mentioned from the list in the interview guide (Table 1). In the present study, we used WHOs standard definition for possible API [15]; The respondents, who presented a plausible description of exposure and reported to have experienced two or more of these symptoms within 48 h of the exposure to pesticides once or several times the past year, were considered to have suffered API.

**Table 1:** Interview guide used on the study of API in Ethiopia

Section	Items in the interview guide	
Socio-demographic information	Identification: House Number: _______; Village: ___________	
	Gender: 1. Male 2. Female	
	Age in years: __________	
	Are you head of the household? (yes/no)	
	How many people live in the family? 1. Male: _____ 2. Female: ____3. Total: ______	
	What is the level of your education in school years? ________________	
Current job	What is your current job?	
	1. Farmer-own land	
	2. Flower farm worker (greenhouse, pack-house, sprayer, other)	
	3. Other (Office worker, small private business, housewife)	
Work experience	How long have you been working in this job?	
Pesticide use	Do you use pesticide at home for pest control? (yes/no)	
	Do you use pesticides on your own farm? (if a farmer) (yes/no)	
	Do they use/spray pesticides in the flower farm? (if you are working in the flower farm) (yes/no)	
Pesticide-related health problems experienced	Have you ever felt health problems within 48 h of exposure to pesticides in the last 12 months?	
	1. Never 2. Once 3. Several times 4. Not exposed to pesticides	
	If you ever had health problem within 48 h of exposure to pesticides, which health symptoms did you experienced? (Tick off all the symptom (s) mentioned by the respondents from the below list)	
	1. Headache	12. Abdominal cramp
	2. Dizziness	13. Chest tightness
	3. Excessive sweeting	14. Dyspnea
	4. Salivation	15. Morning cough
	5. Confusion	16. Day/night time cough
	6. Weakness	17. Shortness of breath
	7. Anxiety	18. Wheezing
	8. Loss of consciousness	19. Miosis
	9. Bradycardia	20. Eye tear
	10. Vomiting	21. Rash on hand
	11. Diarrhea	22. Skin rash
Exposure to pesticides	If you ever had health problem within 48 h of exposure to pesticides, the exposure to pesticides occurred through:	
	1. Pesticide application for household pest control	
	2. Pesticide application at own farm or working at sprayed farm	
	3. While pesticide application or working at sprayed flower farm	
Smoking	Do you currently smoke cigarette (tobacco) daily? (yes/no)	

Five health workers (nurses and public health officers), who were familiar with the area and spoke the local language, did the interview. A half-day orientation/training about the interview guide was given for the interviewers. The interviewers went door-to-door and informed the households about the purpose of the research; and a written consent was obtained. To ensure confidentiality, the interview was done face-to-face with only the interviewer and the respondent present. All participating houses were given numbers for identification instead of participants’ names in order to keep the anonymity of the respondents.

### Statistical analysis

The data were entered into SPSS version 21. Descriptive statistics were used to describe demographic data and the prevalence of API. Chi-square and independent t-tests were used to compare the groups, i.e., living close (<5 kilometers) to the flower farm vs. far away (5–12 kilometers); living close & work in the flower farm vs. living close & don’t work in flower farm as well as flower farm workers vs. all others, for categorical and continuous variables, respectively. Potential confounding variables were all checked for statistical significance between the comparison groups using chi-square and independent t-tests. Those variables with *p* < 0.05, i.e., age, gender, education and being small-scale farmer were all included in the binomial regression model analysis to adjust for these differences, while comparing the API prevalence between the different groups.

## Results

### Characteristics of the population

A total of 516 persons (257 and 259 close and far, respectively) out of the planned 520 participated in the survey (99 % response rate); and out of this, 85 % of the respondents were household heads. The remaining four households did not participate in the survey because they were not available during the interview. The mean age of the surveyed population was 30 years (Table 2). There were significant age differences between the subgroups living close vs. living far away as well as between the subgroups “living close & work” vs. “living close & don’t work” in the flower farm. In terms of current job, 53 % were small-scale farmers, 32 % were flower farm workers and the remaining 15 % were categorized as others such as office work, small business holders and housewives (Table 2). Many of those living close to the flower farms were working at the flower farm (64 %), while the majority of those living far away were small-scale farmers working on their own farms (92 %). The majority of the population (76 %) had a low level of education (primary school level) and those who lived far away from the flower farm had lower education than the others. Only 1 % of the surveyed population smoked cigarettes (tobacco).

**Table 2:** Characteristics of the surveyed population

Variable	Total *N* = 516	Living proximity from FF and work in FF			
		Close to FF	Close & work in FF	Close & don’t work in FF	Far from FF
		*N* = 257	*N* = 164	*N* = 93	*N* = 259
Gender N (%)					
Men	298 (58)	132 (51)	86 (52 %)	46 (49)	166 (64) ^a^**
Women	218 (42)	125 (49)	78 (48)	47 (51)	93 (36)
Family size					
Mean (SD)	4.9 (2.9)	3.6 (2.0)	3.1 (1.9)	4.4 (2.1) ^bb**^	6.4 (2.9)
Age in years					
Mean (SD)	30 (10.6)	26 (6.9)	25 (5.5)	29 (7.9) ^bb**^	34 (12.1) ^b*^
Education in school years					
Mean (SD)	5.5 (3.7)	6.6 (3.5)	6.7 (3.4)	6.4 (3.6)	4.4 (3.6) ^b**^
Type of job N (%)					
Farmer own farm	275 (53)	36 (14)	0	36 (39)	239 (92)
Flower farm	165 (32)	164 (64)	164 (100)	0	1 (0.003)
Green house	87 (17)	86 (34)	86 (52)	0	1 (0.003)
Pack-house	29 (6)	29 (6)	29 (18)	0	0
Sprayer	29 (6)	29 (6)	29 (18)	0	0
Other	20 (4)	20 (4)	20 (12)	0	0
Other	76 (15)	57 (22)	0	57 (61)	19 (7)
Work experience (months)					
Mean (SD)	46 (43)	35 (27)	31 (24.5)	52 (31.3) ^bb^**	71 (61)
Family members working in FF N {%}	109 (21)	104 (20)	82 (50)	22 (22)	5 (1)
Pesticide use N (%) ^c^					
Household use	172 (33)	58 (23)	38 (23)	20 (22)	114 (44) ^d^
Own farm	214 (41)	47 (18)	10 (6)	37 (40) ^dd^**	167 (65) ^d^
Flower farm	173 (34)	172 (67)	157 (96)	15 (16) ^dd^**	1 (0.003) ^d^
Cigarette smoking (tobacco) N (%)	7 (1)	0	0	0	7 (3)

*FF*: flower farm; ^a^: Chi-square test comparing the subgroups close vs. far; ^b^: independent *t*-test comparing the subgroups close vs. far; ^bb^: independent *t*-test comparing the subgroups “close & work” vs. “close & don’t work” in FF; ^c^: percentage may not add to 100 %; ^d^: comparing the subgroups close vs. far by logistic regression; ^dd^: comparing the subgroups “close & work” vs. “close & don’t work” in FF by logistic regression; *:*p* < 0.05; **^:^:*p* < 0.01

The participants used pesticides for pest control in households (mosquito, fleas and bed bug control), crop farming and in the flower farm (33, 41 and 34 % respectively).

### Acute pesticide intoxication (API)

**Fig. 1:**
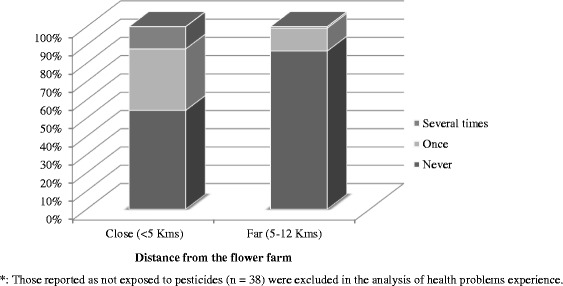
Health problems experienced in the last year by living proximity from the flower farm*

During the last year 29 % (*n* = 141) had experienced health problems within 48 h of exposure to pesticides at least once; 23 % and 6 %, once and several times, respectively (Fig. 1). A total of 136 respondents (26 %) had experienced two or more symptoms, and were thus considered to have had API the last year (Table 3). Among those with API the most frequent self-reported symptoms were nervous system symptoms (79 %) followed by respiratory and gastrointestinal symptoms (58 %), (Table 4).

**Table 3:** Prevalence of API by living proximity from the flower farm and working there or not

	Total	API n (%)	CPR (95 % CI) ^a^	APR (95 % CI) ^b^
Total	516	136 (26)		
Living proximity				
Close	257	107 (42)	3.7 (2.6, 5.4)	3.2 (2.2, 4.8) ^b^
Far (reference)	259	29 (11)	1.0	
Close & don’t work in flower farm	93	15 (16)	1.5 (0.8, 2.9)	1.6 (0.8, 3.2) ^c^
Living close & work in the flower farm or not				
Close & work	164	92 (56)	3.5 (2.1, 5.6)	3.0 (1.8, 4.9) ^b^
Close & don’t work (reference)	93	15 (16)	1.0	
Flower farm worker	165	92 (56)	4.4 (3.3, 6.1)	4.0 (2.9, 5.6) ^b^
Others (reference)	351	44 (13)	1.0	

^a^: Crude Prevalence Ratio; ^b^: Adjusted Prevalence Ratio for age, gender and education; ^c^: Adjusted Prevalence Ratio for age, gender, education and being small-scale farmer; CI: Confidence Interval

**Table 4:** List of self-reported symptoms among the population with API the last year (*N* = 136)

	List of symptoms	Responses *N* (%)
Nervous system	Headache, dizziness, excessive sweeting, salivation, confusion, weakness, anxiety and loss of consciousness	107 (79)
Cardiovascular	Bradycardia	55 (40)
Gastrointestinal	Vomiting, diarrhea, abdominal cramp	79 (58)
Respiratory system	Chest tightness, dyspnea, morning cough, day/night time cough, shortness of breath and wheezing	79 (58)
Ocular	Miosis and eye tear	73 (54)
Dermatologic	Rash on hand and skin rash	69 (51)

Of those living close to the flower farm, 42 % reported to have had API, compared to 11 % among those living far away (PR = 3.7, 95 % CI: 2.6-5.4) (Table 3). The subgroup, who was living close to & worked in the flower farm had a significantly higher proportion of API (56 %) than the subgroup living close to & didn’t work in the flower farm (16 %), (PR = 3.5, 95 % CI: 2.1-5.6). Flower farm workers reported significantly higher API in the last 12 months than “all others”, 56 % & 13 %, respectively (PR = 4.4, 95 % CI: 3.3-6.1). These differences in the prevalence of API between the groups remained significant after adjusting for age, gender and education using binomial regression analyses (Table 3). We found no significant difference in the prevalence of API between the residents living far away and those living close to, but were not working in the flower farm, after adjusting for being small-scale farmer, gender, age and education (Table 3). Pesticide exposure at the flower farm was related to 68 % of the API cases, followed by 16 % and 15 % pesticide exposure related to household pest control and small-scale farmers, respectively. There were significant differences in API prevalence among the different job groups in the flower farm. Greenhouse workers had the highest API prevalence (57 %) followed by sprayers (22 %) and pack-house workers (15 %/) (*p* < 0.01). The prevalence of API among small-scale farmers in the study area was 12 %.

## Discussion

The overall prevalence of symptoms attributable to API in the last 12 months among the households was 26 %, and it was highest in the population, who lived close to and worked at the flower farm.

According to a pilot study done among Ethiopian flower farm workers, the pesticides mostly used in the flower farms were organophosphate, carbamate, pyrethroid +, azole and neonicotinoid [18]. We did not collect information on the type of pesticides involved in our present study as most of the participants had low levels of education, and was expected not to be able to specify the pesticides used. However, the most frequent symptoms reported in the present study are typical symptoms of exposure to the aforementioned pesticides [15].

The prevalence of API among the residents in the present study was similar to the findings in a national survey of male farmers in South Korea on self-reported cases of API (24.7 %; *n* = 1958) [16]. Population-based surveys in Central America (*n* = 32,245) and Nicaragua (*n* = 3169) reported that 2 % and 2.3 % of the population, respectively suffered from API yearly [4, 19]. These low figures compared to our results, is probably explained by the differences in type of studies. The present study should not be considered as a general population study since the majority of the population was selected from an area with a flower farm, and included a high fraction of flower farm workers as well as small- scale farmers. Furthermore, in the studies from Central America and Nicaragua, they asked for self-reported cases of API within the first 24 h of pesticide exposure, while we asked for symptoms within the first 48 h. Also, the differences between these two studies and ours might be attributed to underreporting of cases [20, 21].

The prevalence of API among small-scale farmers (12 %) in our study is slightly higher than reported by Zhang et al. among Chinese farmers (8.8 %; *n* = 910) [14]. This might be due to differences in the definition of API since the Chinese study reported on API cases occurring within 24 h of exposure to pesticides, and not within 48 h as in our study. A survey of agricultural workers in Asian countries also reported lower prevalence of API than our results among small-scale farmers (0.08 % in Indonesia, 2.7 % in Sri Lanka, and 6.7 % in Malaysia; *n* = 8982) [5]. These differences might also be related to the types of pesticides used for the crop they grow in these regions. Since we did not collect specific information on pesticides, it is not possible to verify if this factor accounted for the higher prevalence of API in our study. However, a study among Tanzanian small-scale farmers reported much higher proportion of API (93 %; *n* = 121) than did farmers in our study [22]. The higher prevalence of API in the Tanzanian study might be explained by the difference in methods, as they asked for “past lifetime APP (Acute Pesticide Poisoning) experienced”, while we only asked for their experiences in the past year.

Flower farm workers in the present study also had higher API prevalence (56 %) than in a study of 102 cut-flower workers (23.5 %) in the Philippines [23]. The Philippine study reported on respondents’ illness experiences due to pesticide in the last year, which might explain some of the observed difference.

Our study indicated that residence close to the flower farm as such was not associated with an increased prevalence of API. Thus, it seems unlikely that the pesticides are more accessible among the closest residents. This is in contrast with previous studies that reported increased exposure to pesticides with increased living proximity to farms [24, 25]. In the present study, the increased risk of API among residents living close to the flower farm is associated with being employed at the flower farm since the subgroup living close to the flower farm and working there had significantly more API than those living close but did not work there. This is also supported by the lack of difference in API prevalence between the residents living close to the flower farm but did not work there and those living far away after adjusting for being small-scale farmer. Most of the APIs in the present study were reported to occur after exposure to pesticides at the flower farm. Previous studies have shown that several factors can contribute to increased pesticide exposure for flower-farm workers [17, 22, 25, 26]. Such factors are for instance poor working conditions, inappropriate handling and storage of pesticides, lack of safety training as well as individual’s behavioral factors.

This study demonstrated that the prevalence of API among the residents in the study area is very high. There is very limited information in Ethiopia on the magnitude of API. Our study is located in one region of Ethiopia, but there might be similar problems in other parts of the country. The high prevalence of API seems to be related to pesticide exposure at the flower farm, and decision makers should be informed about the finding and take action to examine this topic further to address this problem in Ethiopia. The handling of pesticides at the flower farms must be improved to avoid API in the future.

### Strength and limitation

Strengths of the present study are that the response rate was high, and we used the WHO’s standard definition of API. However, it is a weakness that there are no objective examinations done of the population, and the severity of the symptoms was not addressed. This may have caused a bias in reporting of API. The information was collected using a population-based survey through interview grouped by proximity to flower farm, in order to include both workers and other persons in the area. This made it possible to obtain information about flower farm workers without entering any workplace. Workplace studies may have the weakness of lack of confidence from the participants, and by the chosen method we presumably increased the likelihood of obtaining correct information also from the workers. There may be re-call bias during the interview, since we asked for symptoms the past year, however, we used interview instruction to minimize the re-call bias. Also, the respondents themselves described symptoms, and they might not have known the name of all symptoms they had. Therefore, the symptom description must be evaluated with caution. Another limitation was that we did not collect detailed information about how intoxication took place and the type of pesticides involved. Thus we suggest further studies to investigate the risk factors of API among the population in the study area.

The use of many interviewers might also be a problem on the consistency of how the interview was done. However, in order to minimize this problem, orientation on the interview guide and interviewing procedures was given to the interviewers by the principal investigator before the actual data collection.

## Conclusion

Our study indicates a 26 % prevalence of self-reported symptoms attributable to API among the population in the study area. The residents living closer than 5 kilometers to the flower farm reported significantly higher prevalence of self-reported API than those living 5–12 kilometers away. This increased risk of API was associated with work at the flower farm.

## Abbreviations

API, acute pesticide intoxication; APP, acute pesticide poisoning; CI, confidence interval; PR, prevalence ratio; SPSS, Statistical Package for the Social Sciences; WHO, World Health Organization
